# Utilizing rewards to dampen fear and its recovery

**DOI:** 10.1038/s41598-025-99758-3

**Published:** 2025-05-21

**Authors:** Elliot C. Brown, Sergio Oroz Artigas, Sarah Elsner, Lu Liu, Soyoung Q. Park

**Affiliations:** 1 Department of Decision Neuroscience and Nutrition, German Institute of Human Nutrition (DIfE), 14558 Nuthetal, Germany; 2 Deutsches Zentrum für Diabetes, 85764 Neuherberg, Germany; 3 Department of Psychology I, University of Lübeck, 23562 Lübeck, Germany; 4 Department of Psychology, Sun Yat-sen University, 510006 Guangzhou, China; 5 Charité-Universitätsmedizin Berlin, Freie Universität Berlin and Humboldt-Universität zu Berlin, Neuroscience Research Center, 10117 Berlin, Germany

**Keywords:** Deepened extinction, Fear reinstatement, Hippocampus, Reward–fear extinction, Skin conductance responses, Amygdala, Extinction, Human behaviour

## Abstract

Fearful memories can be extinguished by repeated exposure, without aversive outcomes. Fear extinction as the basis for exposure therapy, is a common treatment for anxiety and fear-related disorders, but it can be unpleasant and does not always work. In two independent studies, we investigated whether a novel compound extinction procedure (simultaneous presentation of a rewarded and a feared stimulus, reward–fear deepened extinction) attenuates fear while tracking physiological, neural and subjective changes in pleasantness. The reward–fear deepened extinction showed comparable effects to the fear–fear deepened extinction (two-fear compound extinction) in effectively enhancing fear extinction and protecting against the return of fear 1 week later. Moreover, fear extinction accompanying a reward cue was perceived as more pleasant. On the neural level, we identified a network involving hippocampus and amygdala that underlies the benefit of deepened extinction, where connectivity changes also predicted changes in subjective pleasantness. Our study identifies the effects of deepened extinction in regulating long-term fear recovery and its underlying neural mechanism. We shed light on an innovative method to enhance fear extinction, with high translational potential for therapy.

**Supplementary Information:**

The online version contains supplementary material available at 10.1038/s41598-025-99758-3.

## Introduction

Current theories of associative learning emphasize the predictive relationship between the conditioned stimulus and (CS) and the unconditioned stimulus (US). Similar mechanisms are thought to underpin extinction. Extinction is thought to be a learning of a new association, therefore involving error-correction mechanisms^1,2^. Exposure therapy is based upon principles of fear extinction and is one of the most commonly used interventions to treat psychiatric disorders (e.g., anxiety). However, stress, arousal or changes in context^3^ can cause the fear response to return through spontaneous recovery or reinstatement^4^. Although maladaptive fear extinction has been related to several mental illnesses, such as PTSD or phobias^5^, the mechanisms leading to return of fear are still far from being understood. Developing more efficient fear extinction methods will not only help to improve the efficacy of therapeutic interventions but will also provide a deeper mechanistic understanding of extinction and the recovery of fear.

Deepened extinction is a method where two fear cues are presented simultaneously during the extinction phase, and has been shown to powerfully reduce spontaneous fear recovery and reinstatement of fear in both animals and humans, compared to conventional extinction methods^6–11^. Compounding two fear CSs during extinction maximizes learning by increasing the expectancy of a US, thus deepens declining of associative value for the extinguished CS. In other words, compound cues inducing a greater amount of surprise relates to greater expectancy, therefore leading to a higher prediction error, resulting in facilitated extinction^9^. Furthermore, the absence of a fear stimulus (US) might itself exert a rewarding effect mediated by a prediction error signal in the dopaminergic system. Thus the extinction may be strengthened by enhanced dopamine through pairing extinction trials with reward^12^. According to this, the co-presentation of a reward cue together with the fear cue would also effectively extinguish fear. The co-presentation of reward cue might also facilitate basic extinction processes by potentially engaging dopamine system and making the extinction procedure less aversive. Thus, the study would assess the effects of rewards (vs. fear CS) in facilitating fear extinction as well as the mechanisms underlying.

On the neural level, fear conditioning and extinction is thought to primarily involve a network including medial prefrontal cortex (mPFC), amygdala and hippocampus^13^. A history of rodent work has led to a relatively well characterized map of the fear circuit in which the amygdala and hippocampus are thought to store fear memories, while the mPFC integrates information about the CS, being modulated by contextual information from the hippocampus. During extinction, the mPFC is known to inhibit the amygdala which is also modulated by hippocampus^13^. In humans, functional connectivity studies have demonstrated coupling between amygdala, vmPFC and hippocampus during both fear expression and fear extinction^4,14^. Abnormal functioning in the hippocampal–prefrontal–amygdala network has been associated with impaired emotion regulation and post-traumatic stress disorder (PTSD)^15,16^. Though the brain regions are suggested to be involved in fear extinction, no study has yet investigated neural activity related to deepened extinction.

To make the extinction procedure more pleasant and make use of the effectiveness of deepened extinction, we propose an innovative procedure, a reward-based deepened extinction. Here, we simultaneously extincted a reward-conditioned cue together with a fear cue. Across two independent studies, we directly tested the changes during deepened extinction and during reinstatement of fear a week later. Furthermore, the changes in psychophysiological responses and neural processing as a function of the intervention were captured by means of skin conductance responses (SCR) and functional magnetic resonance imaging (fMRI), respectively. We hypothesized that reward-based deepened extinction would be superior to not only conventional extinction, but also to fear-based and no-reward-based deepened extinctions. We also hypothesized that the reward–fear and fear–fear deepened extinctions would show better extinction effect when compared to conventional extinction and no-reward-based deepened extinctions, and the effective deepened extinctions would be superior to prevent the return of fear.

## Materials and methods

### Study 1: skin conductance responses (SCR)

#### Participants

Thirty-five male participants (mean age = 26.3, SD = 4.8) participated in this study. All participants were recruited with posters and online platform from the University of Lübeck (see the Supplement for more details). According to the pilot study investigating the reward effects of different stimuli, only the erotic pictures of women (i.e. an attractive female dressed in lingerie) induced stable SCR in male participants. Based on previous studies using sexual stimuli as a reward, only heterosexual males were included in the study. The experimental procedure included five phases over 2 days with 1 week in between. Fear responses were defined by SCR measurements and only participants who learned the CS–US relationships (i.e. showing higher SCR to the fear (aversive) CS+ than to the fear CS− at the end of conditioning, i.e. in the last run of phase) were considered for further analysis^17,18^, leaving a total of 24 subjects.

This study was approved by the Research Ethics Committee of the University of Lübeck. The experiments were performed in accordance with the Declaration of Helsinki. All participants provided written informed consent prior to participating in the study.

#### Procedures

**Fig. 1:**
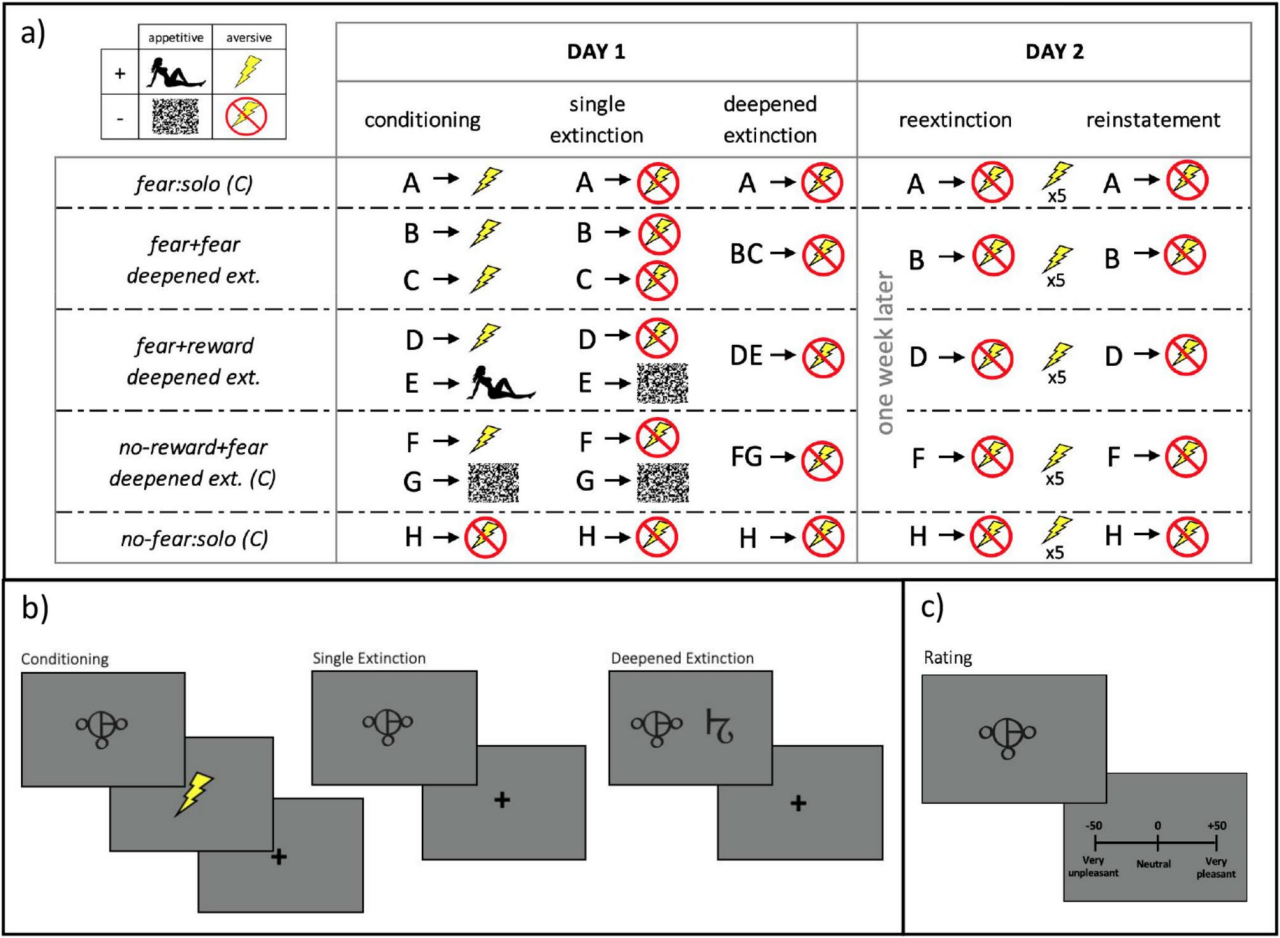
The main part of (**a**) illustrates the design of the experiment with each of the rows representing each of the five conditions or types of cues (A–H) presented on screen in each phase, with columns showing each phase. The first day of testing comprised of conditioning and two extinction phases. During conditioning, cues were paired with an electric shock (fear), an erotic image (reward), scrambled pixels (no-reward), or a no-shock sign (no-fear). In single extinction, cues were presented individually with no outcome (no-fear or no-reward), and in deepened extinction, cues were presented either individually (solo conditions: A, H), or simultaneously with another cue (deepened conditions: BC, DE, FG), all with no outcomes (no-shock sign). A week later, participants were given reextinction and reinstatement phases. To induce return of fear, electric shocks were given randomly before the reinstatement phase. This same design was used across two independent studies, where the first measured only skin conductance responses, and the second collected behavioral and neural data, with subjective ratings of pleasantness and fMRI, respectively. The figure shows in the top left-hand corner of (**a**) the 2 × 2 nature of the presence or absence of appetitive (reward) and aversive (shock) outcomes, and in the main box, all the cues that represented each of the conditions (2 experimental deepened extinction and 3 control “(C)” conditions) and the different testing phases spread over a week of testing; and (**b**) shows examples of stimuli presented on screen for each phase, and (**c**) shows an example of the visual analogue scale used to rate subjective pleasantness of cues in the MRI scanner in the second sample.

The experimental procedure included five phases over 2 days with 1 week in between: On day 1, the participants learned a series of simple cue-outcome associations during (1) classical conditioning, followed by (2) a single extinction and then (3) a deepened extinction phase (Fig. 1a,b). On day 2, subjects underwent (4) a re-extinction and then (5) a reinstatement phase (Fig. 1a) (see the Supplement for more details).

The conditioning phase consisted of 104 trials, in which each of the eight visual cues (abstract symbols) was presented 13 times. CSs+ were paired with either an electric shock or reward with a probability of 38% (5 trials reinforced and 8 not reinforced). For each trial, the CS was presented for 2 s, followed by the US that lasted for 1 s, followed by a fixation cross that stayed on screen for a random duration between 6 and 10 s. Five cues were associated with a shock (labelled A, B, C, D and F in Fig. 1a—aversive CS+, “fear”), one cue with no-shock (labelled H in Fig. 1a—aversive CS−, “no-fear”), one cue with reward (labelled E in Fig. 1a—appetitive CS+, “reward”) and one cue with no-reward (labelled G in Fig. 1a—appetitive CS−, “no-reward”). The phase was split into 4 runs of 26 trials presented in a randomized order.

**Fig. 2:**
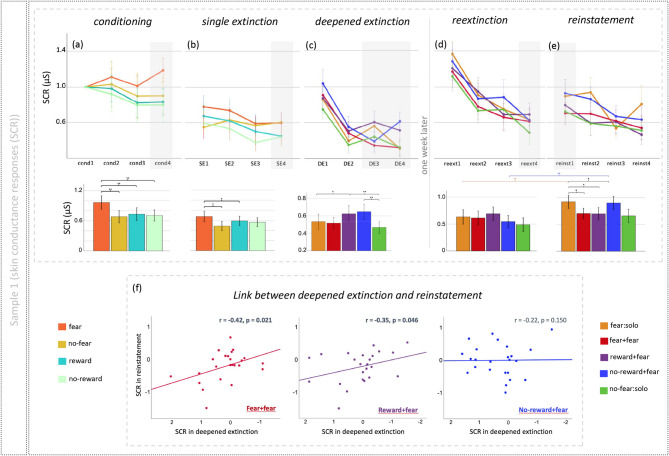
(**a**–**e**) Results of skin conductance responses (SCR) from the first study, with the upper line showing line plots over binned trials, and in the lower line, bar plots of responses taken from trials highlighted in grey. Most importantly, we see in the bar plot below (**e**) that the two experimental deepened extinction conditions (fear + fear and reward + fear) prevent return of fear, whereas the conventional single fear extinction condition and control deepened extinction conditions does not. (**f**) Scatterplots showing the relationship between SCR differences for deepened and conventional single fear extinction cues in reinstatement, and the same SCR differences in deepened extinction. The scatterplots demonstrate a link between deepened extinction and return of fear in reinstatement, only for the two conditions (fear + fear and reward + fear) that were effective in preventing return of fear (*p < 0.05, **p < 0.01).

**Fig. 3:**
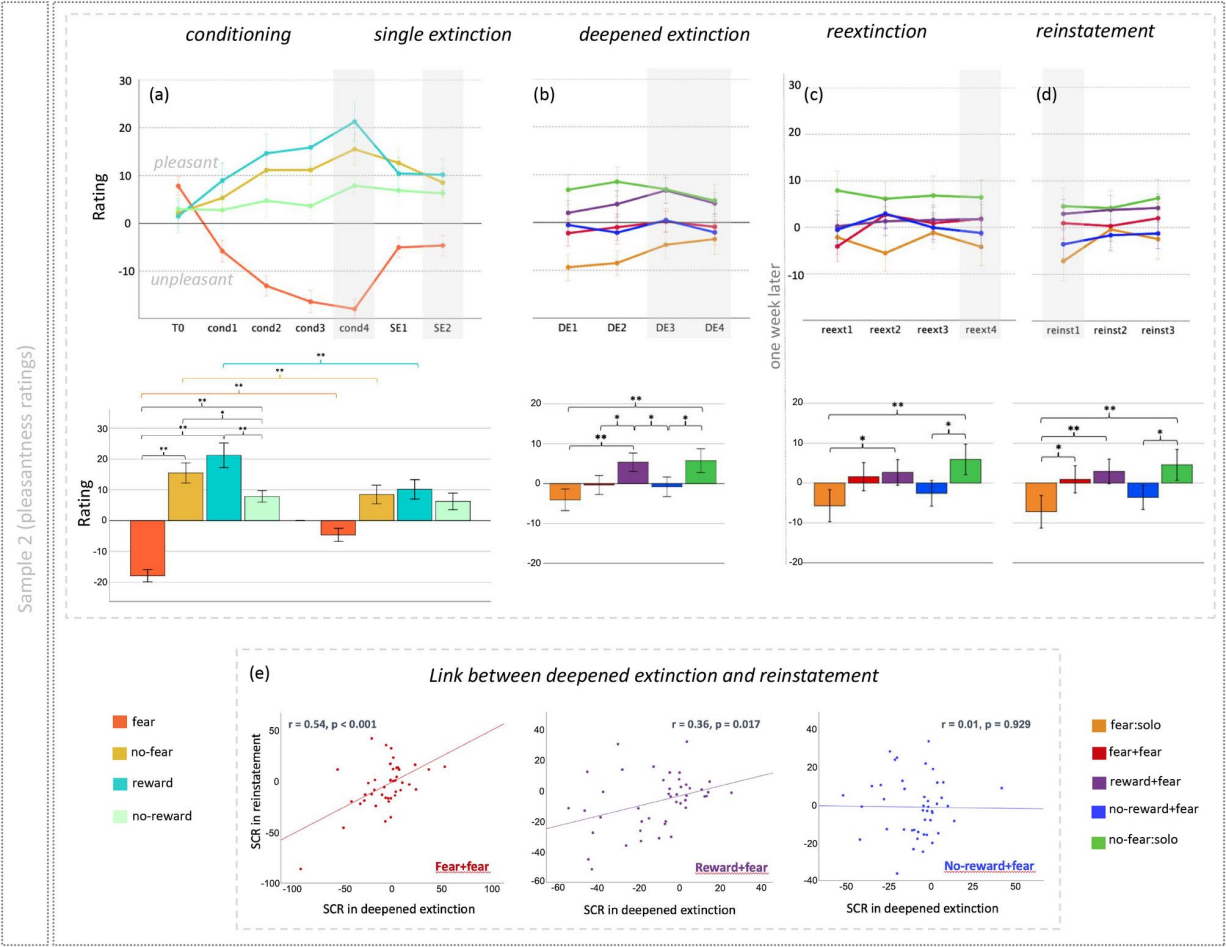
(**a**–**d**) Results of subjective ratings of pleasantness from the second study, with the upper line showing line plots over binned trials, and in the lower line, bar plots of responses taken from trials highlighted in grey. Most importantly, we see in the bar plot below (**b**) that the novel reward-based deepened extinction condition (reward + fear) was preferred over the conventional single fear extinction and other deepened extinction conditions. (**e**) Scatterplots showing the relationship between ratings differences for deepened and conventional single fear extinction cues in reinstatement, and the same ratings differences in deepened extinction. The scatterplots demonstrate a link between deepened extinction and return of fear in reinstatement, only for the two conditions (fear + fear and reward + fear) that were effective in preventing return of fear. Remarkably, this link between phases mirrors those seen in the first study looking at skin conductance responses (*p < 0.05, **p < 0.01).

**Fig. 4:**
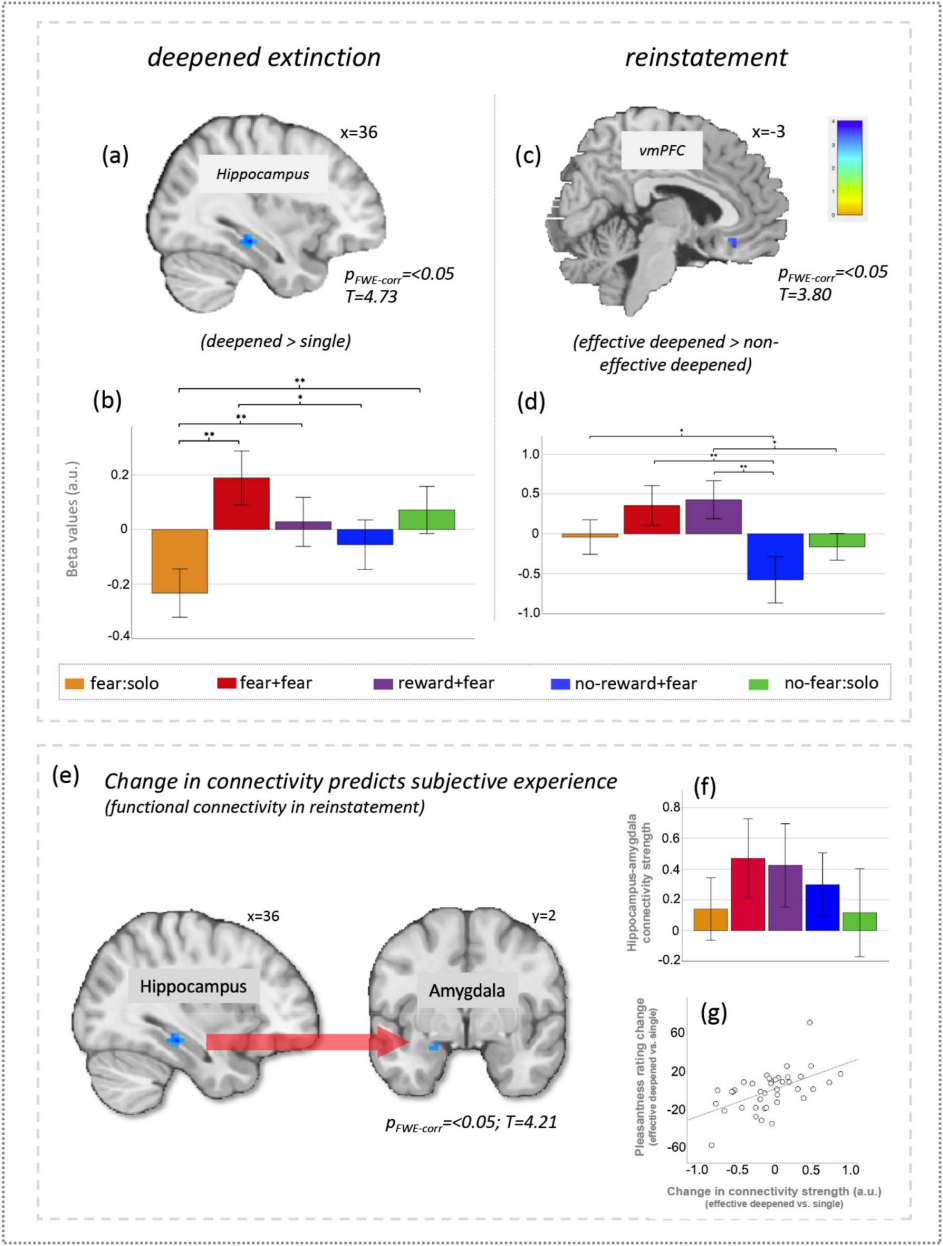
(**a**) During the deepened conditioning phase, we observed significantly greater activation in the hippocampus, when comparing effective deepened extincted versus single extincted cues. (**b**) Extracted beta values from the hippocampus. Both fear-based and reward-based deepened extinction induced significantly greater activation compared to conventional single fear extinction, whereas this difference was not observable for the control deepened extinction cue. We did not observe any significant activation when comparing effective deepened extincted (fear + fear and reward + fear) > control deepened extincted cue (no-reward–fear) (**c**,**d**) A week later, during the reinstatement, the comparison of the effective deepened extincted (fear + fear and reward + fear) > control deepened extincted cue (no-reward–fear), revealed a significantly greater activation in the ventromedial prefrontal cortex (vmPFC). No voxel survived the threshold when comparing effective deepened extinction versus single extinction. (**e**) Results showing functional connectivity during reinstatement (i.e., return of fear) using psychophysiological interactions (PPI) to illustrate a relationship between brain and behavior, linking pleasantness ratings with connectivity. The analysis looked specifically at hippocampus (defined by the deepened extinction effect (**a**) as a seed, which showed significant connectivity with amygdala, as a function of pleasantness in reinstatement, or the return of fear (all results *P*_*FWE-SVC*_ < 0.05). (**f**) Bar plot showing strength of connectivity between hippocampus and amygdala for all conditions, during reinstatement. Pairwise comparisons showed no significant differences between conditions (all *p* > 0.05). (**g**) The scatterplot represents the brain–behaviour relationship showing that a greater change in pleasantness ratings (effective deepened extinction vs. single extinction) was related to a greater change in connectivity strength between hippocampus and amygdala. Values normalized to allow direct comparison (*p < 0.05, **p < 0.01).

The single extinction phase consisted of 64 trials where the same eight cues were presented 8 times in absence of the US (Fig. 1b), as detailed in the Supplement. The second extinction phase consisted of 40 trials under 5 different conditions: (1) extinction control condition—single fear CS (fear:solo—labelled A in Fig. 1a), (2) conventional deepened extinction condition—two fear CSs predicting shock (fear + fear—labelled BC in Fig. 1a), (3) non-conventional reward-based deepened extinction—one reward CS together with one fear CS (reward + fear—labelled DE in Fig. 1a), (4) deepened extinction control condition—no-reward CS together with one fear CS (no-reward + fear—labelled FG in Fig. 1a), and finally, (5) single extinction control condition—no-fear CS alone as reference (no-fear:solo—labelled H in Fig. 1a), as detailed in the Supplement. Several experiments point out that undergoing deepened extinction immediately after conditioning can block rather than enhance extinction^19,20^. For that reason, a transition phase of single extinction was introduced^9,11^. The phases were administered continuously, without a break in between.

On day 2, all aversive CS+s (cue A, B, D, and F in Fig. 1a) and the aversive CS− (cue H in Fig. 1a) were presented alone without the US during both re-extinction (8 presentations for each CS, 40 trials in total) and reinstatement (8 presentations for each CS, 40 trials in total), as detailed in the Supplement. Before starting the reinstatement phase, a train of five unexpected shocks were applied to the participants in order to induce the return of fear (ROF). There were no breaks between re-extinction, reinstatement shocks and the reinstatement phase. The administration or omission of an electrical shock were used as fear or no-fear outcome, respectively. We used an erotic picture as the reward US, and the same image with randomly ordered pixels as the no-reward US. The erotic picture displayed an attractive female dressed in lingerie. The matching of symbols and USs were counterbalanced across participants (see the Supplement for details of the stimuli selection). Paradigm presentation was performed with Cogent2000 toolbox based on MATLAB (MathWorks, Natick, MA, USA).

#### Data acquisition and processing

Skin conductance responses (SCRs) were acquired using a MP150 BIOPAC data acquisition system and AcqKnowledge software (Biopac Systems, Inc., Goleta, CA), and data analysis was performed using MATLAB R2019a (The MathWorks, Natick, MA, USA). SCRs were acquired with a sampling rate of 1000 Hz (see the Supplement for details of SCRs acquisition and processing).

#### Quantification and statistical analyses

##### Conditioning and single extinction phases

As a manipulation check for successful conditioning and single extinction, we performed a 2-way (cue type × phase) repeated-measures ANOVA (rmANOVA) test using SCR data with cue type (fear, no-fear, reward, no-reward) and phase (conditioning, single extinction) as within-subject factors.

##### Deepened extinction phase

Subsequently, we tested whether SCRs during deepened extinction (last half of the trials) differed across conditions. We conducted a rmANOVA with the five conditions (fear:solo, fear + fear, reward + fear, no-reward + fear, no-fear:solo) as a within-subject factor.

##### Re-extinction and reinstatement phase

To test our a priori hypothesis regarding the efficiency of deepened extinction to prevent the return of fear (ROF), we first performed 2-way (condition × phase) rmANOVA using the five conditions (fear:solo, fear + fear, reward + fear, no-reward + fear, no-fear:solo) and phase (re-extinction, reinstatement) as within-subject factors. Following recommendations from previous literature reviews^21,22^, we used the last half trials of the re-extinction phase and the first half trials of the reinstatement phase.

##### Predicting the return of fear (ROF) by deepened extinction

Next, we tested whether deepened extinction successfully prevented the return of fear (ROF). To do this, we calculated the individual differences in SCR responses between the signal cue (fear:solo) and compound cues (fear + fear, reward + fear, no-reward + fear) by using the first half trials of deepened extinction (salient response) and the first half trials of reinstatement (sustained responses) for each cue condition (see the Supplement for details). The first half trials of deepened extinction were used to capture the underlying process that occurred during the initial absence of US representing the process rather than the outcome of extinction. Spearman correlation analyses were applied.

### Study 2: subjective ratings and functional neuroimaging (fMRI)

In the second experiment using a completely independent sample from the first, we aimed to understand the underlying psychological and neural mechanisms behind deepened extinction. For this, we assessed the subject’s report of the CSs+ (pleasantness rating) as well as neural fMRI data.

#### Participants

Forty-seven male subjects (mean age = 25.6, SD = 3.7) participated in this study. Consistent with study 1, only participants that showed learning according to subjective ratings (i.e., rating the fear cue as less pleasant than the no fear cue in the last run of the conditioning phase) were included. The study was approved by the local Medical Ethical Commission of the University of Lübeck, and participants provided informed consent. Exclusion criteria were any history of neurological or psychiatric disorders. Five subjects were excluded due to excessive movement (> 3 mm) and 2 of them uncompleted the 2nd testing day.

#### Procedures

The experimental procedure and timing of the trials were analogous to experiment 1 and detailed in the Supplement. We used Psychtoolbox-3^23,24^ based on MATLAB (MathWorks, Natick, MA, USA) for stimulus presentation. The stimulation electrodes that delivered the shock were changed to MRI-compatible 1.5 mm bipolar electrodes with a 5 kOhm resistance (Easycap, Herrsching, Germany).

#### Behavioral data acquisition

Learning of fear and reward, as well as reinstatement effects were measured by subjective ratings in the MRI scanner. Each cue was presented in a randomized order on screen for 2 s, followed by a Visual Analog Scale (VAS) to rate each of the eight cues according to their pleasantness on a scale from very unpleasant to very pleasant (− 50 to + 50, 0 = neutral, Fig. 1c). The VAS stayed on screen until participants made a response. Ratings were done at the beginning of the experiment and end of each run, making a total of 11 ratings on day 1, and 7 on day 2. In order to correct for inter-individual differences, ratings were rank-corrected for each subject for days 1 and 2 separately.

##### Behavioral analyses

All statistical analyses performed with subjective ratings were analogous to the analyses done on the SCR data from experiment 1. Spearman correlation analyses were also used to test the prediction of deepened extinction to reinstatement.

#### fMRI analysis

##### MRI data acquisition

The scanning was performed on a 3 T Magneton Skyra scanner (Siemens, Erlangen, Germany) using a 64‐channel head coil. Functional data were acquired using a repetition time (TR) of 2 s, echo time (TE) of 30 ms and a Flip angle of 90° (see the Supplement for details).

##### fMRI data preprocessing

All fMRI analyses were done using Statistical Parametric Mapping software 12 (SPM12, Wellcome Department of Imaging Neuroscience, London, UK) in MATLAB R2018b (MathWorks, Natick, MA, USA). The functional imaging data was spatially realigned, slice time corrected, inspected for excessive motion (scans with displacement ≥ 3.0 mm were excluded), spatial normalized to the standard Montreal Neurological Institute EPI template (voxel size = 3 × 3 × 3 mm^3^), and smoothed with an isometric Gaussian kernel (full width at half-maximum of 8 mm).

##### Statistical analysis of functional data

Statistical analysis of individual participant imaging data was performed using first-level fixed-effects analyses using general linear model (GLM)^25^. To identify the brain regions specifically involved in the effective deepened extinction, two GLM were estimated to investigate the fear extinction effects (the brain responses during deepened extinction phase) and the brain responses for the return of fear (ROF) effects during reinstatement, respectively. For the deepened extinction phase, the regressors set up for statistical first-level (single-subject) analysis were: one regressor per extinction type (solo cue conditions: A, H; deepened double cue conditions: BC, DE, FG) with onset times set to the presentation of the cues (CSs). For the reinstatement phase, the regressors set up for statistical first-level (single-subject) analysis were: one regressor per fear conditioned cues (the fear CSs: A, B, D, F, H) with onset times set to the presentation of the cues. Timing of task events for was convolved with the canonical hemodynamic response function, with the realignment parameters included as regressors of no interest. To remove low-frequency signal drift, a high-pass filter (128 Hz) was applied. For each subject, contrast images were constructed between extinction types and between fear CSs to examine regional brain activation related to fear extinction and reinstatement, respectively.

Region of interest (ROI) analyses was conducted. ROI definitions for anterior cingulate cortex (ACC), dorsolateral prefrontal cortex (DLPFC), ventromedial prefrontal cortex (VMPFC), orbitofrontal cortex (OFC), hippocampus, amygdala, thalamus, nucleus accumbens (NAcc), and insula were taken based on previous literature on fear learning and fear extinction^26,27^ and according to the Harvard–Oxford anatomical atlas^28–31^. ROIs were thresholded to 70% to prevent overlap and the inclusion of non-brain areas. Correction for multiple comparison was performed at an α-level of *p* < 0.05 with our pre-defined ROIs and using small volume correction (SVC) according to Gaussian random field theory and the family-wise error rate method (corrected by means of GRFT with voxel-level *p* < 0.001 and cluster-level *p* < 0.05)^25^. We also report significant brain activation at the whole-brain level corrected by means of GRFT with voxel-level *p* < 0.001 and clusterlevel *p* < 0.05 to result in a family-wise error rate of 5%. This method follows from previous literature on fear extinction that performed a similar analysis^32^.

##### Deepened extinction phase

To identify the brain regions specifically involved in the effective deepened extinction, a contrast image of fear:solo cue > the two compound cues (i.e., fear + fear, reward + fear cues; these were effective in reducing the return of fear in experiment 1) was constructed for each subject. In addition, we also conducted a contrast to identify regions related to effective deepened extinction (i.e. [fear + fear and reward + fear] vs. [no-reward + fear]). These contrast images were entered into a second level random-effects analysis using one-sample t-test design to investigate treatment effects.

##### Reinstatement phase

Another GLM was conducted to identify brain regions related to the return of fear (ROF) effects during reinstatement for solo versus compound cues. Onset regressors per run were defined following the same criteria as the analysis done for deepened extinction. Two contrast images were defined using (1) the fear:solo cue > the two compound cues (fear + fear, reward + fear), and (2) the two effective compound cues (fear + fear and reward + fear) > the non-effective compound cues (no-reward + fear]). These contrast images were entered into a second level random-effects analysis using a one-sample t-test design to investigate treatment effects.

##### The brain–behavior relationship

To uncover the functional connectivity modulated by the effectiveness of deepened extinction during reinstatement and its relationship with subjective experience of deepened extinction, we performed a whole-brain psycho-physiological interaction (PPI) analysis and a regression analysis^33,34^. The PPI analyses were conducted to assess the functional connectivity changes between the seed region and other brain regions in different extinction conditions. Specifically, we conducted a first-level PPI GLM contrast at the individual level using the hippocampus seed (a cluster survived from contrast effective vs. solo fear extinctions in deepened extinction phase: 9 voxels, X = 36, Y = − 28, Z = − 13) to extract the physiological regressor, and modeled compound and solo conditions in both deepened extinction and reinstatement phases as stick functions to extract psychological regressors. At the second-level a one-sample t-test was conducted to identify the regions coupling with hippocampus associated with the effective extinction during ROF. Furthermore, the PPI regressors that resulted from the first-level analysis were put into a group-level GLM contrast, using individual pleasantness rating changes (effective > solo fear extinction cues) as regressor.

## Results

### Study 1: physiological responses to a novel reward-based deepened extinction

Twenty-four participants (mean age = 27.2, SD = 3.6) who learned the CS–US relationships were included in statistical analyses. Successful fear conditioning was found to result in a higher SCR to the fear CS+s than to all other cue types (main effect of cue: *F*(1,23) = 7.37, *p* < 0.001, partial η^2^ = 0.537; Fig. 2a). A reduction in SCR was also seen for the fear CS+ from the conditioning to single extinction phase (main effect of phase: *F*(1,23) = 10.19, *p* = 0.004, partial η^2^ = 0.307). In the deepened extinction phase we found a main effect of condition (*F*(1,23) = 3.94, *p* = 0.005, partial η^2^ = 0.537). Specifically, the novel reward–fear deepened extinction elicited a greater SCR than the conventional single fear extinction cue (*t*(23) = 2.32, *p* = 0.030; Fig. 2c), whereas the fear–fear deepened extinction cue showed no difference to the single fear cue. In Day 2 one week later, during reinstatement phase, both the novel reward–fear and fear–fear deepened extinction cues exhibited significantly lower SCRs compared to the single fear extinction cue (*t*(23) = 2.24, *p* = 0.035; *t*(23) = 2.31, *p* = 0.030, respectively; Fig. 2e), suggesting the effectiveness in protecting against the ROF. During reextinction phase, there were no significant differences between conditions at the beginning of reextinction (Fig. S1). For all results, see Fig. 2a–e and Fig. S1 for line and bar plots, Tables S1 and S4 for means and statistics, and the Supplementary material for details.

### Study 1: the link between physiological responses in deepened extinction and reinstatement

Significant correlations between deepened extinction and reinstatement for SCRs in the (effective) fear-based (*r* = − 0.42, *p* = 0.021) and reward-based (*r* = − 0.35, *p* = 0.046) deepened extincted cues, but not for the (non-effective) no-reward–fear (*r* = − 0.22, *p* = 0.150) deepened extincted cue (Fig. 2f) (see the Supplement for more details). Comparison of *r*-values using Fisher’s *r*-to-*Z* transformation indicated no significant correlation differences between fear–fear and no-reward–fear deepened extincted cues, nor between reward–fear and no-reward–fear deepened extincted cues. The results suggest that for the effective deepened extincted cues, the greater the SCR responses during the deepened extinction phase, the better the fear was extincted during reinstatement.

### Study 2: subjective experiences and underlying neural mechanisms of a novel reward-based deepened extinction

In the second independent study, naïve male participants (N = 47 total, included in fMRI analysis: N = 42 on first day, N = 40 on second day) underwent the same experimental paradigm while their brain data was collected by means of fMRI. As expected, the fear CS+ was less pleasant than all other cues during conditioning (main effect of cue: *F*(1,45) = 25.47, *p* < 0.001, partial η^2^ = 0.361; Fig. 3a). During the transition from the conditioning to extinction phases, we observed changes in pleasantness for the fear and reward cues (interaction effect of cue × phase: *F*(1,45) = 16.70, *p* < 0.001, partial η^2^ = 0.271; Fig. 3a), showing both successful conditioning and single extinction of fear in subjective feelings. During the deepened extinction phase, the novel reward–fear deepened extinction cue was considered to be more pleasant than the single fear extinction cue, fear–fear and no-reward–fear deepened extinction cues (for single fear cue: *t*(44) = 3.85, *p* > *0.001*; for fear–fear cue: *t*(44) = 2.09, *p* = 0.04; for no-reward–fear cue: *t*(44) = 2.23, *p* = 0.03), and was comparable to the no-fear control condition (*t*(44) = − 0.12, *p* = 0.91; main effect of condition: *F*(4,44) = 4.30, *p* = 0.002, partial η^2^ = 0.089; Fig. 3b). During reinstatement 1 week later, the fear cue, that underwent reward–fear deepened extinction, was again rated as significantly more pleasant than the conventional single fear extinction cue (*t*(43) = 2.90, *p* = 0.006; Fig. 3d). The fear–fear deepened extinction also showed significantly higher pleasant than the single fear condition during reinstatement test (*t*(43) = 2.10, *p* = 0.04; Fig. 3d). Consistent with Study 1, the deepened extinction control condition using no-reward showed no difference to the conventional single fear extinction cue during reinstatement test, implying the inhibition effect of reward–fear and fear–fear deepened extinction on ROF (Fig. 3a–d for line and bar plots, and Tables S2 and S5 for means and statistics).

### Study 2: the link between subjective experiences of deepened extinction and reinstatement

A correlation analysis identified that participants with greater pleasantness during deepened extinction reported greater pleasantness during reinstatement, suggesting a successful protection against fear return. Importantly, this link was significant only for the effective deepened-extinction cues (fear–fear: *r* = 0.54, *p* < 0.001, and novel reward–fear: *r* = 0.36, *p* = 0.017 deepened extinction conditions; no-reward–fear condition: *r* = 0.01, *p* = 0.929) (Fig. 3e). Comparison of *r*-values using Fisher’s *r*-to-*Z* transformation indicated significant correlation difference between fear–fear and no-reward–fear deepened extincted cues (*Z* = 2.79, *p* = 0.005), and marginal significant correlation difference between reward–fear and no-reward–fear deepened extincted cues (*Z* = 1.72, *p* = 0.085).

### Study 2: the underlying brain network for deepened extinction

We focused on the changes in brain response for effective deepened extinction during the deepened extinction and reinstatement phases. In deepened extinction phase, the contrast [solo fear cue > (fear–fear and reward–fear extinction cues)] showed that the solo fear cue elicited greater activation in posterior hippocampus (x = 36, y = − 28, z = − 13; *t* = 4.73; *P*_*FWE-SVC*_ = 0.01; Fig. 4a,b and Tables S3, S6). No other ROI regions was survived in the contrast between effective deepened extinction and single fear extinction.

During reinstatement, we compared the effective and non-effective deepened extinction ([fear–fear and reward–fear deepened extinction cues] vs. no-reward–fear deepened extinction cue), and found greater activation in the vmPFC (x = − 3, y = 29, z = − 16; *t* = 3.80; *P*_*FWE-SVC*_ = 0.03; Fig. 4c,d). We also conducted whole-brain analysis to compared the brain responses to deepened extincted cues versus single cues in the reinstatement phase, for the results, see Fig. S2 and Table S7 and the Supplementary material for details.

In addition, analyses to contrast fear + fear, reward + fear and no-reward + fear for both the deepened extinction and reinstatement phases were performed and we found no significant results for either phase. We also analyzed the single extinction phase, contrasting all CS+s with all CS−s and found no significant results within the predefined ROIs.

### Study 2: amygdala–hippocampus connectivity reflects differences in deepened extinction and its efficacy on fear extinction

The PPI analysis identified that the right hippocampus region (identified as the deepened extinction area) showed stronger connectivity with the left amygdala in response for effective extinction cues processing compared with signal fear cues during reinstatement phase (*P*_*FWE-SVC*_ = 0.02; Fig. 4e,f). Furthermore, by linking the PPI result to individual differences in pleasantness changes (effective deepened-extinction cues vs. single-extinction cue), we found that the change in hippocampus–amygdala connectivity was significantly positively correlated with changes in subjective ratings (Fig. 4g). It was important to note here that the correlations for the ROI analyses could not survive the correction for multiple comparisons, using a Bonferroni corrected *p* value of *p* < 0.006.

## Discussion

Across two different studies with independent samples, the data presented here show powerful evidence that our deepened extinction procedure (especially the innovative reward–fear deepened extinction) can be utilized to empower fear extinction not only by making the procedure more pleasant, but also by preventing the return of fear effectively 1 week later. In an independent sample, the reward-based deepened extinction also reduced SCR over typical single fear extinction during reinstatement, making it a desirable candidate for clinical translation. We also identified a link between the beneficial effects of deepened extinction and fear reinstatement for effective compound cues, though this was only visible in subjective ratings and in neural functions in the second sample. On the neural level, we identified a network involving hippocampus and amygdala that underlies the benefit of this novel deepened extinction, where connectivity changes also predicted changes in subjective pleasantness. Taken together, we present converging evidence at the behavioral, physiological and neural levels that the underlying mechanism behind the protective effects of deepened extinction. We identify a candidate brain circuit responsible for the beneficial effects of deepened extinction, whereby protection against fear recall occurred through the modulation of amygdala via the hippocampus^35,36^.

The mechanisms underlying the two effective deepened extinction procedures may be different. Previous studies have implied that the benefits of deepened extinction over single extinction come from the error-correction theory^9^. It defines learning (in both acquisition and extinction) as the discrepancy between what is expected to occur and what actually occurs. The presence of a second stimulus (fear + fear cue) appears to enhance excitation and so to enhance the discrepancy from nonreinforcement, yielding a greater error term that promotes further associative loss. Our study revealed a better prevention of ROF by applying fear–fear deepen extinction may attribute to the decremental association due to the greater error signal induced by the compound fear CS cues. The result also consistent with one cross-species study about the deepen extinction against the post-extinction recovery of fear^37^. Furthermore, the innovative reward–fear deepen extinction manipulation may exert its effect by recruiting the reward-related dopaminergic system^38^. However, results on the neural level identified no reward network significantly involved in the reward–fear compound extinction or reinstatement phase. An alternative interpretation of our results is that the beneficial effects of deepened extinction could have been possibly driven by salience. Studies investigating memory have suggested that emotionally aroused stimuli were more likely to be remembered than neutral ones independent on whether they are positive or negative^39,40^. Consistently, our findings may implicate that these memory processes were driven by amygdala responses no matter learned-cues were positive or negative^41^. Considering studies have shown that the salience network can modulate SCRs to arousing stimuli^42^, our findings that the reward–fear deepened extinction elicited greater SCRs compared to single extinction imply that the compound extinction cues with opposite valence may be more salient. The benefit of the deepened extinction effect in our study was also closely tied to amygdala-hippocampus connectivity implicated to be involved in the processing of salient events^35^. The findings also shed new light on anxiety and fear-related disorders (e.g., post-traumatic stress disorder). In addition to fear-related exposure and extinction, reward-related CS and reward–fear deepened extinction could also be considered. Reward related outcomes such as positive social interaction, recovered resources following the traumatic situation may exert effects by recruiting the reward-related dopaminergic system and/or by increasing the salience of the situation to make the extinction procedure less aversive. Thus, further research is needed to transform the related deepened extinction approaches to the treatment of fear-related disorders.

Recent human neural imaging meta-analyses have identified an extended fear conditioning network, including the dorsal anterior cingulate cortex, ACC, PCC, DLPFC, vmPFC, OFC, hippocampus, amygdala, thalamus, and insula when comparing typical single extinction to the effective deepened extinction cues^26,27^. Our imaging results showed greater activation in hippocampus involved in effective deepened extinction (vs single fear extinction) processing during the deepened extinction phase, and greater activation in dorsal ACC, PCC, vmPFC, and insula in the same contrast during reinstatement, areas of which related to typical fear conditioning. The hippocampus may be more engaged in re-learning the associations for deepened extincted cues than single extincted cues during the extinction phase^43^. Furthermore, the changes (effective > single deepened extinction) in hippocampus–amygdala connectivity predicted pleasantness changes during reinstatement, implicating the role of the hippocampus–amygdala network as a function of effective deepened extinction in protecting against the subjective experience return. In addition, during reinstatement, we found that the vmPFC showed increased activation for the effective (prevented ROF) deepened extincted cues than the non-effective (did not prevent ROF) deepened extincted cues, the area of which has been proposed to play a crucial role in new associative learning and fear extinction, coding the retrieval of extinction^13^. Taken together, the findings revealed underlying mechanism behind the effective deepened extinction as well as the reinstatement, suggesting possible approaches for further clinical intervention.

There are some limitations to this study. First, there is currently no consensus on the criteria for determining whether fear learning occurred across participants, and relying on one index such as SCR may not be ideal^44^. Second, the lack of a no-reward sign, but only the presence of a no-shock sign in the deepened extinction phase was another limitation. This may influence the extinction of the reward CS−. Furthermore, if the reward itself was enhancing extinction, then presentation of a reward concurrently with the onset of the CS could be another component of the experimental design for future studies to take into consideration. In addition, the Study 2 was lack of SCR measurement during the fMRI scanning, which made the study lack of enough physiological arousal index. The combined acquisition of SCR psychophysiological and fMRI data will be conducted in future studies. A final limitation of this study was our inclusion of only male participants. Further research is needed to determine the effects of deepened extinction in regulating fear recovery in females.

## Conclusions

Our results systematically demonstrate whether and how rewards can be powerfully utilized to enhance extinction and protect against the return of fear. The deepened extinction procedure could empower fear extinction not only by making the procedure more pleasant, but also by preventing the return of fear and reducing SCR over typical single fear extinction during reinstatement. We further identify a candidate brain circuit responsible for the beneficial effects of deepened extinction, whereby protection against fear recall occurred through the modulation of amygdala via the hippocampus. The application of a reward-based deepened extinction could optimize therapy by increasing its benefits, making it more pleasant, therefore may not only increase efficacy, but also improve patient engagement.

## Electronic supplementary material

Below is the link to the electronic supplementary material.

Supplementary Material 1

## Data Availability

The behavioral and skin conductance responses (SCRs) data are available on an OSF repository (https://osf.io/u4sfp/). The fMRI statistical maps are available on a NeuroVault repository (https://neurovault.org/collections/LLMBQXJJ/). All study related materials in the paper are available on request to S.Q.P. or L.L.
