# Supplementary material for: Utilizing rewards to dampen fear and its recovery
Source: Sci Rep. 2025 May 21;15:17671. doi: 10.1038/s41598-025-99758-3 (PMC12095787; doi:10.1038/s41598-025-99758-3)
Supplement: Supplementary file 1 — Supplementary Material 1 [file 41598_2025_99758_MOESM1_ESM.doc]

**Utilizing rewards to dampen fear and its recovery**

**Supplementary Materials**

**Supplementary Methods and Materials**

We designed an experiment comparing a variety of forms of single and deepened extinction using different cues associated with feared (electric shocks) and rewarding (erotic images) outcomes within a subject (Fig 1). The experimental procedure began with a conditioning phase with aversive CS+ (fear), aversive CS- (no fear), appetitive CS+ (reward) and appetitive CS- (no reward) cues. This was followed by a single extinction phase, and then a deepened extinction phase with 5 conditions: 1) conventional single fear extinction (“fear:solo”), 2) fear-based deepened extinction (“fear+fear”; increased salience), 3) reward-based deepened extinction (“reward+fear”; increased salience), and two control conditions: 4) control no-reward-fear deepened extinction (“no-reward+fear”; no increased salience), 5) no-fear single extinction (“no-fear:solo”). One week later, participants returned to undergo a spontaneous recovery phase where they were presented with previously extincted cues from each of the 5 conditions, and then a reinstatement phase preceded by random shocks in order to assess return of fear.

**Study 1 - Skin conductance responses (SCR)**

**Participants**

All participants received 24 Euros as financial compensation for their participation in the study.

**Procedure**

Before the experimental procedure, all participants underwent a shock intensity calibration. Here, participants received electrical stimulation on the left hand between the thumb and index finger of increasing intensity starting from 2.5 mA for a fixed time of 200 msec. The intensity was then increased stepwise by 0.5 mA and subjects had to indicate when they considered the intensity as “the maximum acceptable pain”. The shock level was set to the reported intensity limit (mean ± SD, 8.77 ± 3.30 mA) for the administration of the experimental procedure. Electric stimulation was applied with a DS5 stimulator (Digitimer, Welwyn Garden City, United Kingdom) and platinum pin electrodes (WASP electrodes, Specialty Developments, Bexley, United Kingdom).

For each trial of the single extinction phase, the CS was presented for 2 seconds, followed by a fixation cross that stayed on screen for a random duration between 7 and 11 seconds. Trials were spread over 2 runs where each cue was presented four times in each run in a randomized order. In each trial of the deepened extinction phase, the CS was presented for 2 seconds, followed by a fixation cross that stayed on screen for a random duration between 7 and 11 seconds. Cues were presented in a random order of the deepened extinction phase. Each condition corresponds to each row in Figure 1a.

On day 2 one week later, all aversive CS+s and the aversive CS- were presented alone without the US during both re-extinction and reinstatement. In each trial of the re-extinction and reinstatement phases, the CS was presented for 2 seconds, followed by a fixation cross that stayed on screen for a random duration between 7 and 11 seconds. The re-extinction and reinstatement phases were both split into four runs each, where each cue was presented twice in a random order in each run. We used an erotic picture as the reward US, and the same image with randomly ordered pixels as the no-reward US. This picture displayed an attractive female dressed in lingerie. The image was chosen based on ratings from male members of the lab and from colleagues who were asked to choose the most rewarding image from a set, and the one that was most highly chosen was the image that was used in our study. The administration or omission of an electrical shock were used as fear or no-fear outcome, respectively. In order to maintain attention during the paradigm, participants were instructed to click the left mouse button using their dominant hand as soon as they saw the cue (i.e. the CS) in all phases of the experiment, but they were told that their performance would not influence the outcome.

**Data acquisition and processing**

Skin conductance responses (SCRs) were acquired with a sampling rate of 1000 Hz. Two disposable AgCl electrodes were placed on the distal phalanx of the index and middle fingers of the non-dominant hand for the recording of the skin conductance.

For SCR processing, the following steps were applied: SCRs were measured as the difference between the averaged signal 0-2 secs after the cue onset and the peak signal between 2-8 secs ^6,7^, including only non-reinforced trials ^8^. Differences smaller than 0.02 µS were considered as no response ^9,10^. We applied a square-root transformation for normalization of data ^5^.

**Quantification and statistical analyses**

**Predicting the return of fear (ROF) by deepened extinction.** Correlation analyses were applied to SCR compound-solo differences at the start of deepened extinction with the SCR compound-solo differences at the start of reinstatement in order to investigate whether responses during deepened extinction were related to responses in reinstatement.

**Study 2 – Subjective ratings and functional neuroimaging**

**Procedures**

Before scanning began, a shock intensity calibration procedure was also performed (mean = 8.40 SD = 4.74 mA; no significant difference between experiment 1 and experiment 2: t(80) = 0.39, p = 0.68), just as described in experiment 1. In the task used during scanning, the conditioning phase consisted of 104 trials (8 cues repeated 13 times) divided in 4 runs, but CSs+ were paired with the shock or reward with a 46% contingency (6 trials reinforced and 7 not reinforced). The conditioning was followed by 48 trials of a single extinction phase (6 trials per cue) divided in 2 runs and 80 trials of a deepened extinction phase (16 trials per cue combination) in 4 runs. One week later, participants underwent the re-extinction and reinstatement phases in the scanner. During re-extinction, each cue was presented 12 times, making a total of 60 trials distributed in 3 runs. Consecutively, five unexpected shocks were applied, followed by another 60 trials of reinstatement (12 trials 5 cues) divided in 3 runs.

**Behavioral analyses.** We first checked for successful conditioning and extinction. We used rmANOVAs to compare participants’ subjective ratings for all conditions against each other. Spearman correlation analyses were used to test whether effects for ratings in deepened extinction were related to ROF in reinstatement, as done in experiment 1.

**fMRI analysis**

**MRI data acquisition.** The scanning was performed on a 3T Magneton Skyra scanner (Siemens, Erlangen, Germany) using a 64‐channel head coil. Functional data were acquired using a repetition time (TR) of 2s, echo time (TE) of 30 ms and a Flip angle of 90º. Each volume contained 68 slices with a voxel size of 3 x 3 x 3 mm, covering the whole brain. A T‐1 structural scanning was additionally performed for the purpose of anatomical localization (160 slices, voxel size of 1×1×1 mm, TR 1900 ms, TE 2.44 ms and 9° flip angle). Images were displayed by using an MRI compatible LCD monitor situated in the back of the scanner (Nordic NeuroLab, Bergen, Norway) with an active area of 698x393 mm. Decisions were recorded through response button device on the right hand (Neuroelectrics, Philadelphia, PA, USA).

**Statistical analysis of functional data.** All fMRI analyses were done using SPM12 in MATLAB R2018b (MathWorks, Natick, MA, USA). Each run was considered as a different session and time courses were extracted for each condition and for each phase separately. Onset times were set to the presentation of the cues (CSs). Six movement parameters were determined for each run separately and used as regressors of no interest in each phase.

We selected and made the mask of the vmPFC ROI according to previous literature (Lonsdorf et al., 2014). Specifically, coordinate was set to [x, y, z = 0, 40, -12] and the coordinate was used as the center of a box of dimensions 20×16×16 mm that equally covered both hemispheres (Raczka et al., 2010; Paret et al., 2011).

For both the deepened extinction and reinstatement phases, planned comparisons were performed to compare all conditions. This was done with mean beta values for each condition at the individual level that were extracted with functional ROIs derived from the clusters showing significant differences seen in contrasts at the group level.

**The brain-behavior relationship.** In order to be in line with the SCR analysis, the first trials of deepened extinction were used to capture the neural processing that occurred during the initial absence of US representing the process of extinction rather than the outcome effect of extinction.

**Supplementary Results**

**Study 1: Physiological responses to a novel reward-based deepened extinction**

In Day 2 one week later, no differences were found in SCRs across all conditions during re-extinction, including the no-fear and no-reward-fear control conditions, suggesting that all conditions were resistant to spontaneous recovery. In Day 2 one week later, during reinstatement, the no-reward-fear deepened extinction showed comparable SCRs when compared to the single fear extinction cue (*p*>0.05; Fig 2e), which was therefore considered as non-effective condition in preventing the ROF. The results are the first evidence for a saliency-driven mechanism underlying reward-based deepened extinction. It is also important to note that the reward-based deepened extinction was as effective as fear-based deepened extinction. Our results clearly demonstrate that the reward-based deepened extinction was more effective in extinguishing fear than conventional single fear extinction or no-reward-based deepened extinction and was comparable to the fear-based deepened extinction.

**The link between physiological responses in deepened extinction and reinstatement**

The results suggest that for the effective deepened extincted cues, the greater the SCR responses during the deepened extinction phase, the better the fear was extincted during reinstatement. However, when we compared correlation coefficients across conditions, using a Fisher-Z-transformation and an α-level of 0.05, we did not see significant differences. Furthermore, these correlations would not have survived correction for multiple comparisons, using a Bonferroni corrected p-value of *p*<0.017.

**Study 2: Subjective experiences and underlying neural mechanisms of a novel reward-based deepened extinction**

For the self-report pleasantness, the results suggested that reward-based deepened extinction was consistently preferred not only over conventional fear extinction, but also fear-based deepened extinction in extinguishing fear, although the two deepened extinctions were comparably powerful in reinstatement.

**The link between subjective experiences of deepened extinction and reinstatement**

The correlation analysis about participants’ pleasantness during deepened extinction and pleasantness during reinstatement showed no significant correlation for non-effective cue (no-reward-fear condition, *r*=0.01, *p*=0.929) (Fig 3e). Furthermore, by comparing correlation coefficients in effective and non-effective deepened extinctions using the Fisher-Z-transformation, we found significant difference between the fear-based and no-reward-fear conditions. The significant correlation results in effective deepened extinction conditions also survived multiple comparisons correction using a Bonferroni corrected p-value of 0.017.

**The underlying brain network for deepened extinction**

In deepened extinction phase, we performed additional contrasts to compare solo fear with reward+fear and no-reward+fear, solo fear with fear+fear and no-reward+fear, and solo fear with all compound cues, and found no significant effects in any of these contrasts. Therefore, we can rule out that the effects were only driven by the difference between single and compound cues.

Furthermore, the comparison of fear-based vs. reward-based deepened extinction conditions showed no significant differences in brain activity during deepened extinction phase. During reinstatement, no ROI was survived in the effective extinction contrast [(fear-fear and reward-fear extinction cues) > solo fear cue]. However, a whole-brain analysis found the effective extinction contrast elicited greater activation across typical fear conditioning regions including anterior cingulate cortex (ACC), posterior cingulate cortex (PCC), medial prefrontal cortex (mPFC), insula and precentral gyrus (Fig S2 and Tables S3, S6 and S8).

**Tables**

**Table S1.** Description of skin conductance responses for all phases and stimulus types, from first sample of participants

| **phase** | **stimulus type** | **mean (𝜇s)** | **SD (𝜇s)** |
| --- | --- | --- | --- |
| **conditioning** | fear | 0.965 | 0.661 |
|  | no fear | 0.682 | 0.601 |
|  | reward | 0.732 | 0.627 |
|  | no reward | 0.707 | 0.545 |
| **single extinction** | fear | 0.676 | 0.524 |
|  | no fear | 0.487 | 0.441 |
|  | reward | 0.588 | 0.464 |
|  | no reward | 0.562 | 0.439 |
| **deepened extinction** | fear:solo | 0.532 | 0.427 |
|  | fear+fear | 0.514 | 0.328 |
|  | reward+fear | 0.642 | 0.443 |
|  | no-reward+fear | 0.649 | 0.415 |
|  | no-fear:solo | 0.466 | 0.345 |
| **reextinction** | fear:solo | 0.691 | 0.649 |
|  | fear+fear | 0.636 | 0.633 |
|  | reward+fear | 0.690 | 0.637 |
|  | no-reward+fear | 0.755 | 0.745 |
|  | no-fear:solo | 0.616 | 0.507 |
| **reinstatement** | fear:solo | 0.918 | 0.594 |
|  | fear+fear | 0.701 | 0.486 |
|  | reward+fear | 0.695 | 0.564 |
|  | no-reward+fear | 0.897 | 0.599 |
|  | no-fear:solo | 0.658 | 0.598 |

**Table S2.** Description of subjective pleasantness ratings taken with a visual analogue scale (VAS), from second sample of participants

| **phase** | **stimulus type** | **mean (VAS)** | **SD (VAS)** |
| --- | --- | --- | --- |
| **conditioning** | fear | -17.776 | 13.346 |
|  | no fear | 14.923 | 22.241 |
|  | reward | 20.723 | 27.300 |
|  | no reward | 7.586 | 12.877 |
| **single extinction** | fear | -4.637 | 14.458 |
|  | no fear | 8.503 | 20.464 |
|  | reward | 10.167 | 21.570 |
|  | no reward | 6.270 | 18.179 |
| **deepened extinction** | fear:solo | -4.059 | 18.110 |
|  | fear+fear | -0.347 | 15.939 |
|  | fear+reward | 5.360 | 15.434 |
|  | no-reward+fear | -0.796 | 16.425 |
|  | no-fear:solo | 5.755 | 19.975 |
| **reextinction** | fear:solo | -5.127 | 25.579 |
|  | fear+fear | 1.273 | 23.269 |
|  | fear+reward | 2.663 | 21.100 |
|  | no-reward+fear | -2.086 | 21.360 |
|  | no-fear:solo | 5.779 | 24.966 |
| **reinstatement** | fear:solo | -7.197 | 27.093 |
|  | fear+fear | 0.925 | 22.589 |
|  | fear+reward | 2.920 | 20.513 |
|  | no-reward+fear | -3.591 | 20.138 |
|  | no-fear:solo | 4.569 | 25.597 |

**Table S3.** Means and SDs for fMRI beta values extracted from regions that showed significant effects for the contrast single vs. deepened extinction

| **phase** | **ROI** | **stimulus type** | **mean (beta)** | **SD (beta)** |
| --- | --- | --- | --- | --- |
| **deepened extinction** | hippocampus | fear:solo | -0.254 | 0.576 |
|  |  | fear+fear | 0.168 | 0.642 |
|  |  | fear+reward | 0.007 | 0.583 |
|  |  | no-reward+fear | -0.077 | 0.592 |
|  |  | no-fear:solo | 0.051 | 0.559 |
| **reinstatement** | ACC | fear:solo | 0.472 | 1.440 |
|  |  | fear+fear | 1.513 | 1.332 |
|  |  | fear+reward | 1.202 | 1.157 |
|  |  | no-reward+fear | 1.288 | 1.470 |
|  |  | no-fear:solo | 1.147 | 1.501 |
|  | PCC | fear:solo | 0.546 | 0.948 |
|  |  | fear+fear | 1.269 | 1.213 |
|  |  | fear+reward | 1.006 | 1.043 |
|  |  | no-reward+fear | 0.991 | 1.232 |
|  |  | no-fear:solo | 0.754 | 1.178 |
|  | vmPFC | fear:solo | -0.174 | 1.617 |
|  |  | fear+fear | 0.828 | 2.032 |
|  |  | fear+reward | 0.706 | 1.561 |
|  |  | no-reward+fear | 0.629 | 1.678 |
|  |  | no-fear:solo | 0.668 | 1.962 |
|  | insula | fear:solo | 0.608 | 0.801 |
|  |  | fear+fear | 1.260 | 0.933 |
|  |  | fear+reward | 1.034 | 0.851 |
|  |  | no-reward+fear | 0.992 | 1.135 |
|  |  | no-fear:solo | 0.819 | 1.201 |
|  | thalamus | fear:solo | 0.736 | 1.674 |
|  |  | fear+fear | 1.578 | 1.607 |
|  |  | fear+reward | 2.045 | 2.277 |
|  |  | no-reward+fear | 1.374 | 2.064 |
|  |  | no-fear:solo | 1.233 | 1.725 |

**Table S4.** Main results from statistical tests for skin conductance responses, showing only significant effects (p<0.05), from first sample of participants

**Table S5.** Main results from statistical tests for subjective pleasantness ratings, showing only significant effects (p<0.05), from second sample of participants

**
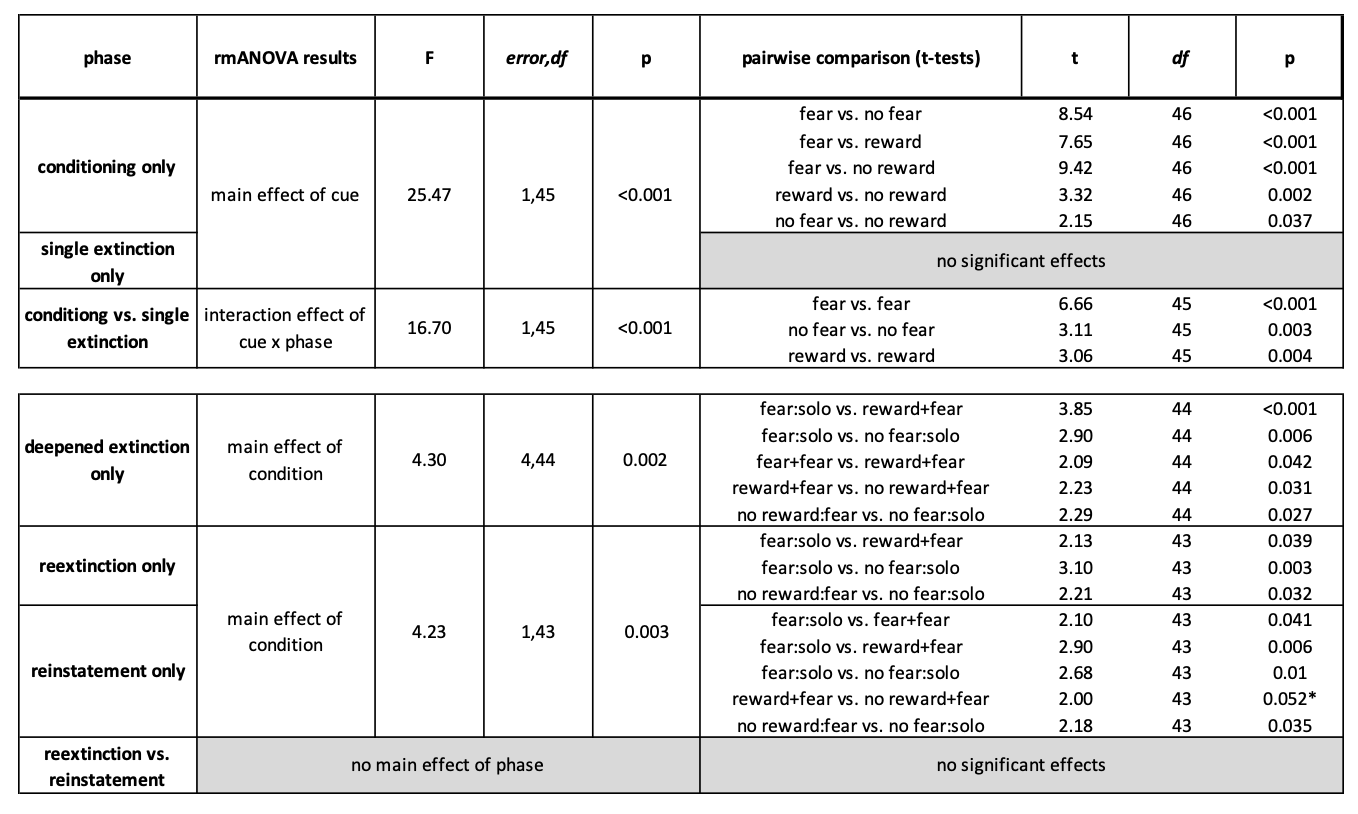
**

**Table S6.** Main results from fMRI contrasts, showing significant effects (*p*<0.05), from second sample of participants

**
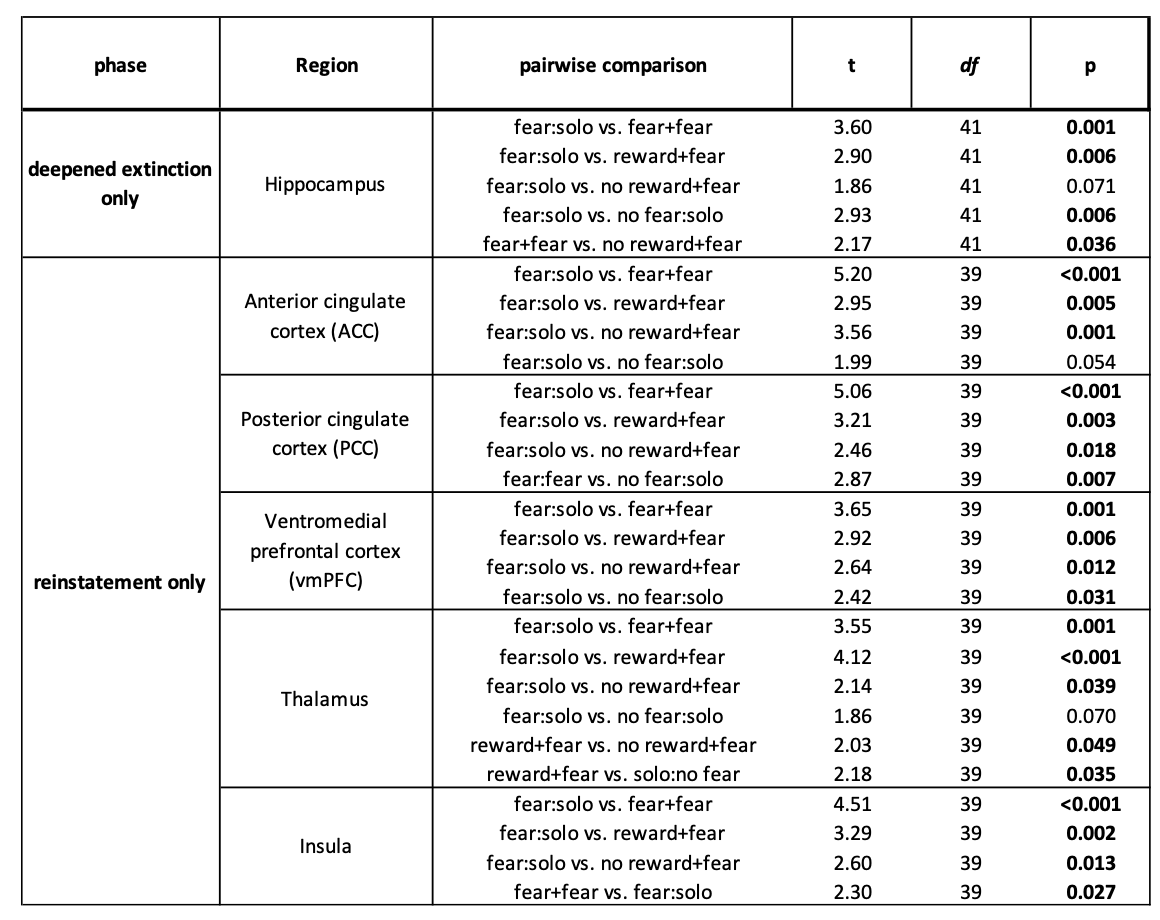
**

**Table S7.** fMRI region of interest (ROI) results from the contrast of deepened vs. single extincted cues during the deepened extinction (*P_FWE-SVC_* < 0.05); and whole-brain analysis results from the effective vs. single cue conditions during reinstatement phases (*P_FWE_* <0.05).

| **ROI** | **Hemis-phere** | **voxels** | **T** | **MNI coordinates (peak)**  **x y z** |
| --- | --- | --- | --- | --- |
| *Deepened Extinction* | | | | |
| hippocampus | R | 9 | 4.73 | 36 -28 -13 |
| *Reinstatement* | | | | |
| ACC/mPFC/dmPFC | R | 354 | 4.40 | 0 35 5 |
| precentral gyrus/ postcentral gyrus/ IPL | R | 380 | 4.58 | 60 2 11 |
| precentral gyrus/ postcentral gyrus | L | 184 | 4.42 | -48 -4 26 |
| insula/ STG/ SMG | R | 143 | 4.55 | 45 -34 20 |
| insula/ SMG/ IPL/ STG | L | 299 | 4.85 | -39 -19 17 |
| PCC/ MCC/ SMA/ precuneus | R/L | 367 | 5.19 | -15 -37 50 |
| postcentral gyrus/ precentral gyrus | L | 118 | 4.63 | -24 -31 74 |

**Figures**


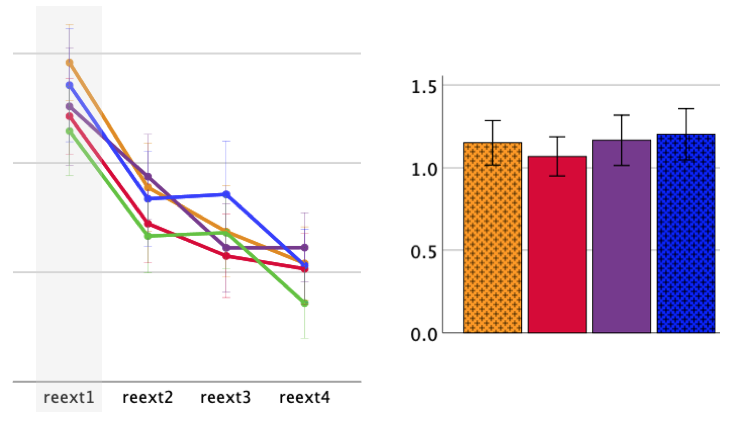


**Figure S1**. Skin conductance response results (μS) for spontaneous recovery showing that there are no significant differences between conditions at the beginning of reextinction, one week after conditioning and deepened extinction phases.


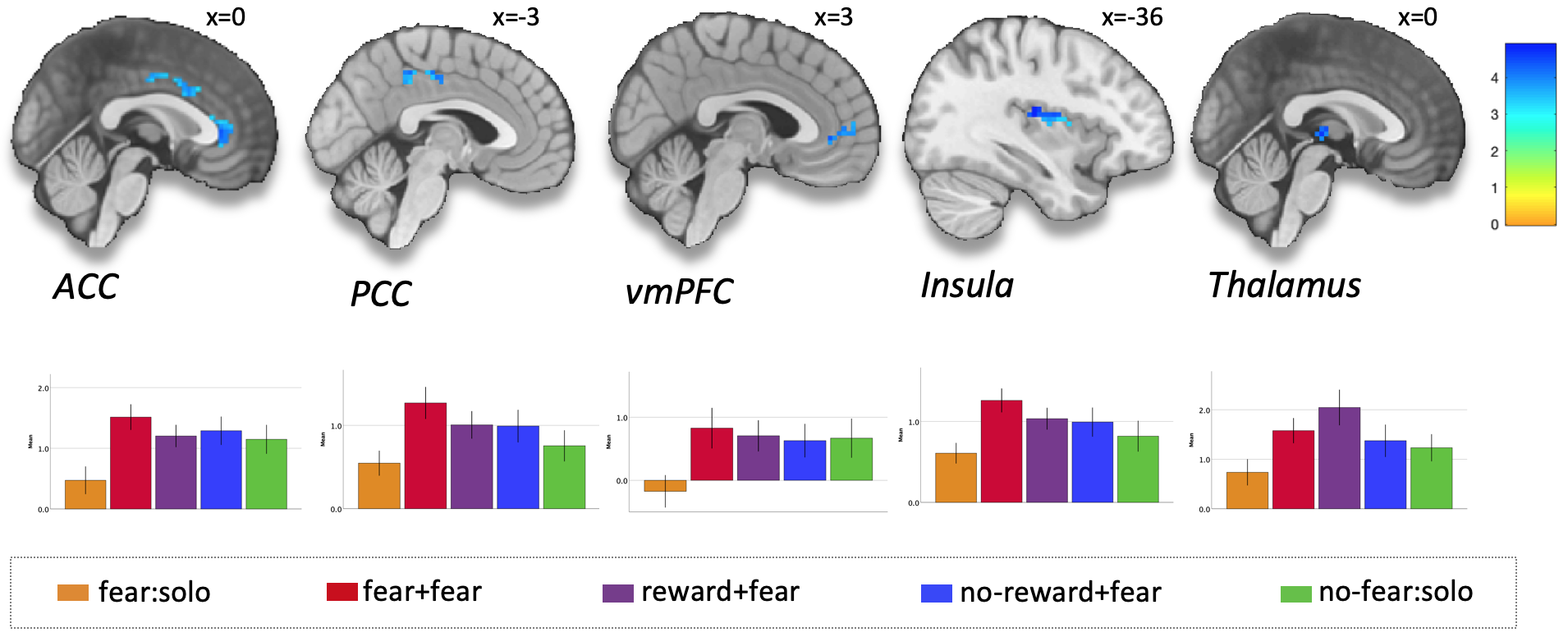

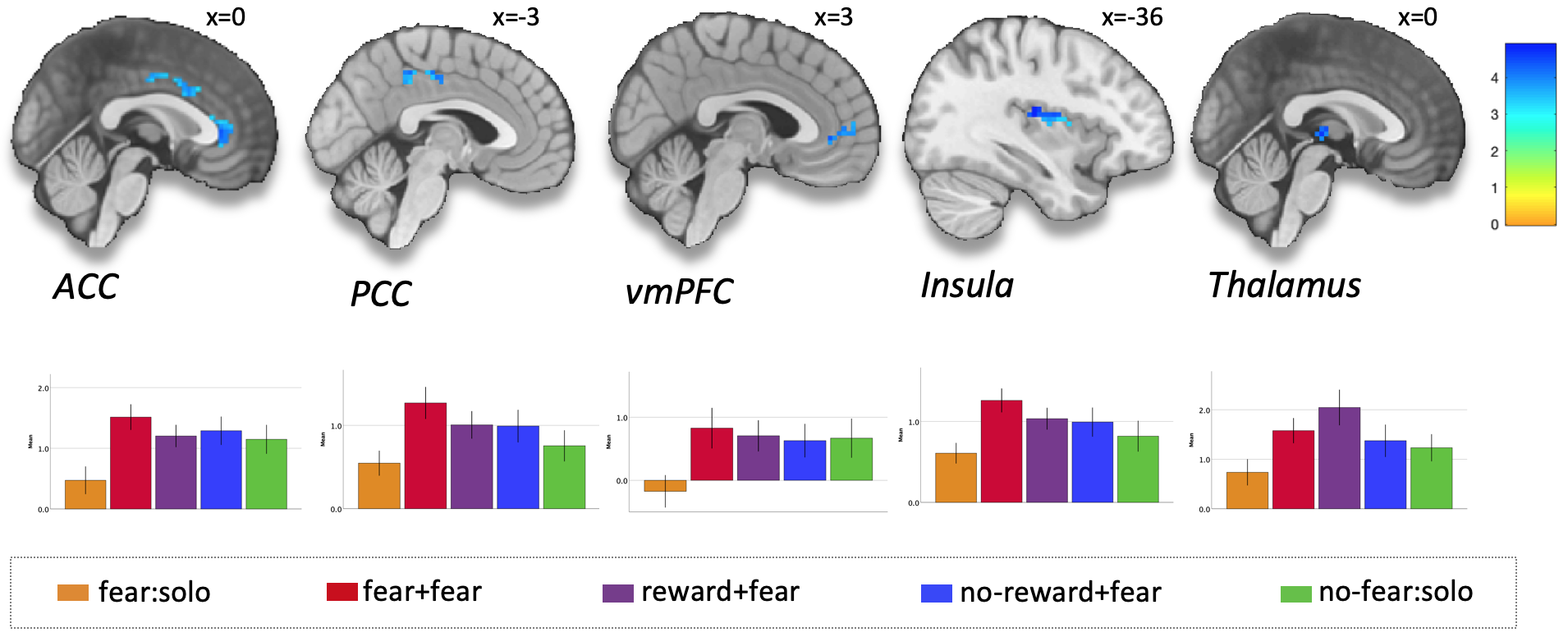

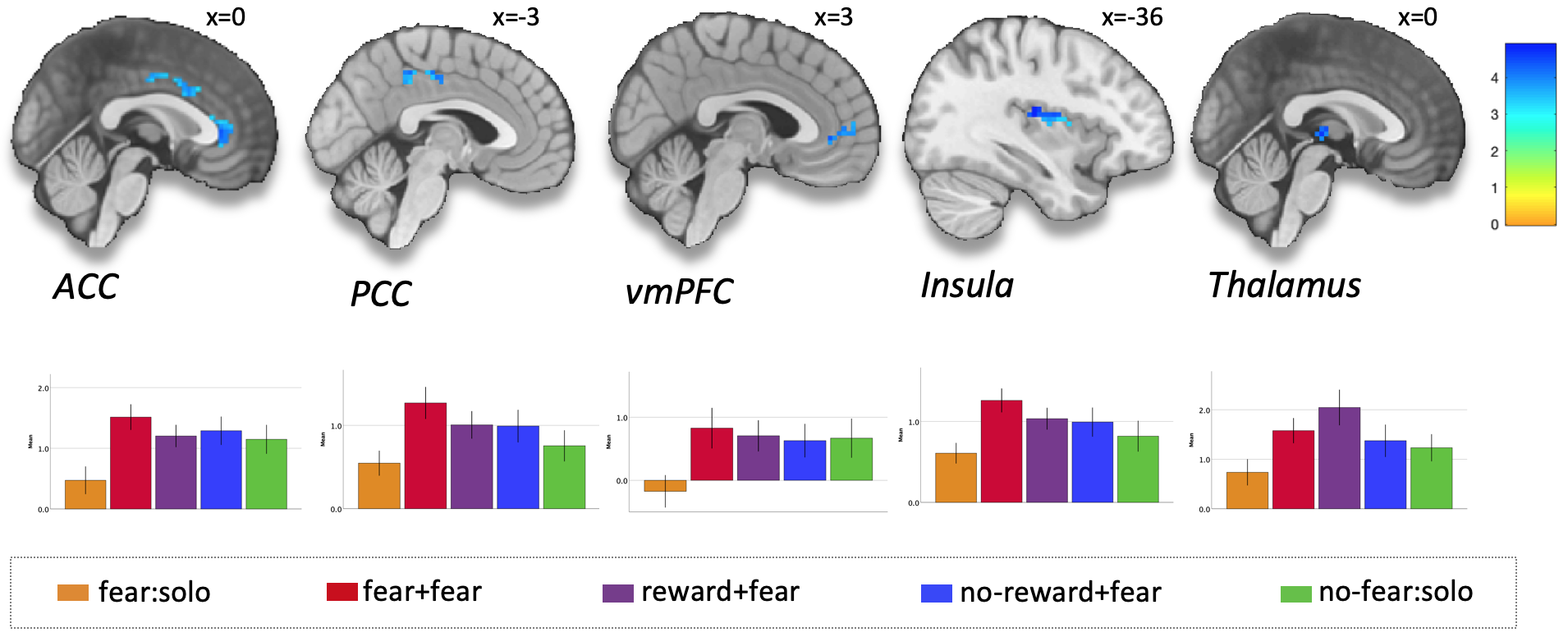


**Figure S2.** condition-comparison of brain responses to deepened extincted cues > single cues in the reinstatement phase (*P_FWE_*<0.05)

**References**

1. Inslicht, S. S. *et al.* Sex differences in fear conditioning in posttraumatic stress disorder. *J Psychiatr Res* **47**, 64–71 (2013).

2. Pineno, O., Zilski, J. M. & Schachtman, T. R. Second-order conditioning during a compound extinction treatment. *Learn Motiv* **38**, 172–192 (2007).

3. Vervliet, B., Vansteenwegen, D., Hermans, D. & Eelen, P. Concurrent excitors limit the extinction of conditioned fear in humans. *Behav Res Ther* **45**, 375–383 (2007).

4. Rescorla, R. A. Deepened extinction from compound stimulus presentation. *J Exp Psychol Anim Behav Process* **32**, 135 (2006).

5. Culver, N. C., Vervliet, B. & Craske, M. G. Compound extinction: Using the Rescorla--Wagner model to maximize exposure therapy effects for anxiety disorders. *Clin Psychol Sci* **3**, 335–348 (2015).

6. Klucken, T., Kruse, O., Schweckendiek, J. & Stark, R. Increased skin conductance responses and neural activity during fear conditioning are associated with a repressive coping style. *Front Behav Neurosci* **9**, 132 (2015).

7. Raes, A. K. & De Raedt, R. The effect of counterconditioning on evaluative responses and harm expectancy in a fear conditioning paradigm. *Behav Ther* **43**, 757–767 (2012).

8. Bulganin, L., Bach, D. R. & Wittmann, B. C. Prior fear conditioning and reward learning interact in fear and reward networks. *Front Behav Neurosci* **8**, 67 (2014).

9. Schiller, D., Kanen, J. W., LeDoux, J. E., Monfils, M.-H. & Phelps, E. A. Extinction during reconsolidation of threat memory diminishes prefrontal cortex involvement. *Proc Natl Acad Sci* **110**, 20040–20045 (2013).

10. Andreatta, M. & Pauli, P. Appetitive vs. aversive conditioning in humans. *Front Behav Neurosci* **9**, 128 (2015).

11. Brainard, D. H. The psychophysics toolbox. *Spat Vis* **10**, 433–436 (1997).

12. Pelli, D. G. & Vision, S. The VideoToolbox software for visual psychophysics: Transforming numbers into movies. *Spat Vis* **10**, 437–442 (1997).

13. Friston, K. J. *et al.* Psychophysiological and modulatory interactions in neuroimaging. *Neuroimage* **6**, 218–229 (1997).

14. Park, S. Q. *et al.* A neural link between generosity and happiness. *Nat Commun* **8**, 1–10 (2017).
